# Fractal dimension and the navigational information provided by natural scenes

**DOI:** 10.1371/journal.pone.0196227

**Published:** 2018-05-07

**Authors:** Moosarreza Shamsyeh Zahedi, Jochen Zeil

**Affiliations:** 1 Research School of Biology, Australian National University, Canberra ACT, Australia; 2 Department of Mathematics, Payame Noor University, Tehran, Iran; University of Sussex, UNITED KINGDOM

## Abstract

Recent work on virtual reality navigation in humans has suggested that navigational success is inversely correlated with the fractal dimension (FD) of artificial scenes. Here we investigate the generality of this claim by analysing the relationship between the fractal dimension of natural insect navigation environments and a quantitative measure of the navigational information content of natural scenes. We show that the fractal dimension of natural scenes is in general inversely proportional to the information they provide to navigating agents on heading direction as measured by the rotational image difference function (rotIDF). The rotIDF determines the precision and accuracy with which the orientation of a reference image can be recovered or maintained and the range over which a gradient descent in image differences will find the minimum of the rotIDF, that is the reference orientation. However, scenes with similar fractal dimension can differ significantly in the depth of the rotIDF, because FD does not discriminate between the orientations of edges, while the rotIDF is mainly affected by edge orientation parallel to the axis of rotation. We present a new equation for the rotIDF relating navigational information to quantifiable image properties such as contrast to show (1) that for any given scene the maximum value of the rotIDF (its depth) is proportional to pixel variance and (2) that FD is inversely proportional to pixel variance. This contrast dependence, together with scene differences in orientation statistics, explains why there is no strict relationship between FD and navigational information. Our experimental data and their numerical analysis corroborate these results.

## Introduction

Navigation is a vital ability for humans and animals and it relies on spatial representations of their environments. On a local scale, vision provides reliable navigational information on heading direction, routes and locations which under natural conditions has been most thoroughly studied in insects. One of the most influential concepts originating in insect navigation research is the snapshot hypothesis, which was derived from classical experiments on bees [1] and ants [2]. According to this hypothesis, insects store panoramic snapshots when looking back at goal locations such as the nest or a food source and then are able to return to such goals by deriving navigational instructions from a comparison between current views with remembered snapshots. It has subsequently been shown that panoramic image differences develop smoothly with distance from a reference location (translational image differences: transIDF) in natural environments and in addition provide robust local visual compass information (rotational image differences: rotIDF) [3–7].

The navigational information provided by the visual panorama can thus be quantified and mapped for different environments [8] although the scene properties that determine the available information are only partly understood: the shape and depth of transIDFs depend on the depth structure of the environment [4], i.e. the distribution of objects, basically because image differences due to translation are generated by distance-dependent motion parallax in translational optic flow. In contrast, rotIDFs are generated by rotational optic flow and their shape and depth are independent of the depth structure, but dependent on the spatial frequency composition of scenes [4]. The range over which panoramic images provide navigational information on location through gradient descent in translational image differences thus depends on the depth structure of habitats with transIDFs being narrower and having a steeper gradient in densely cluttered, compared to open habitats [3–4]. The range over which panoramic images provide local compass information, i.e. the distances from a reference location at which there still is a detectable minimum of the rotational image difference function depends on the degree to which the distant panorama (which contributes low spatial frequencies to panoramic images) remains visible. The more cluttered the scene is with nearby objects that obscure distant landscape features, the smaller is the range over which a panoramic image can provide compass information [7–8].

At this stage, both experimental and simulation work on insect navigation paints the following picture on how insects at least make use of panoramic images for following routes and for pinpointing goals [8–9]: ants, bees and wasps perform learning walks and flights when first leaving their nest or a newly discovered food source during which they pivot around the goal while repeatedly looking back at it from different compass directions [10–17]. In the case of ants, such directed views across the goal can—depending on habitat structure–provide guidance as to the direction of the goal from up to 15 m away, a range over which the rotIDF has a detectable minimum when current views are compared with views close to the nest that were memorised during learning walks [6,8,13]. The behaviour of ants when they are released 10 m away from the nest at locations they are unlikely to have been before indicates that they are making use of this information [7,13,14]: they systematically scan the scene first in one direction, then in the other direction before heading in the direction of their nest. The ants’ gaze during scanning is not directed at salient features in the scene, but would allow ants to determine the orientation in which there is a minimum between a stored snapshot and the current scene. Walking into the direction of that minimum, ants will encounter values of the rotIDF minimum that become smaller and smaller as they approach the goal, because the value of that minimum is the value of the transIDF at that location. In this sense, then, mapping the range over which the rotIDF relative to a reference snapshot still has a detectable minimum is a way of mapping the range over which a gradient descent in image differences will end up in the reference location.

In the following analysis, we will be using the rotIDF in outdoor environments as one of the ways in which navigational information can be quantified, but add the caveat that this may not be the most adequate way of doing so when considering human navigation, because humans are likely to make much more use of object recognition than insects [18–19]. However, our main interest here is to test whether fractal dimension as a measure of scene complexity is also a useful measure of the navigational information provided by natural scenes.

Fractal geometry has been used to describe natural and built environments but has only recently been related to navigational performance: Juliani et al. [20], tested human navigation in virtual reality environments and found that navigational performance is inversely related to the fractal dimension of artificial scenes. Natural environments often display complex visual patterns that are irregular, repeat at increasingly fine size scales and are best described using fractal geometry [21–22]. The fractal dimension (FD) is a central construct developed in fractal geometry to describe the geometric complexity of natural phenomena as well as other complex forms [23]. In the modelling literature there has been no clear consensus on how best to measure and manipulate environmental complexity in a generalizable way when assessing human navigation performance [20].

In this paper, we will investigate the navigational information provided in five different natural scenes at different times of day and its relationship with fractal dimension. We will show that there is an inverse relationship between the variance of pixel values and the fractal dimension of an image and an inverse relationship between the depth of rotIDFs and fractal dimension. We corroborate our results with experimental data.

## Materials and methods

### The fractal dimension (FD) of natural panoramic images

**Fig 1:**
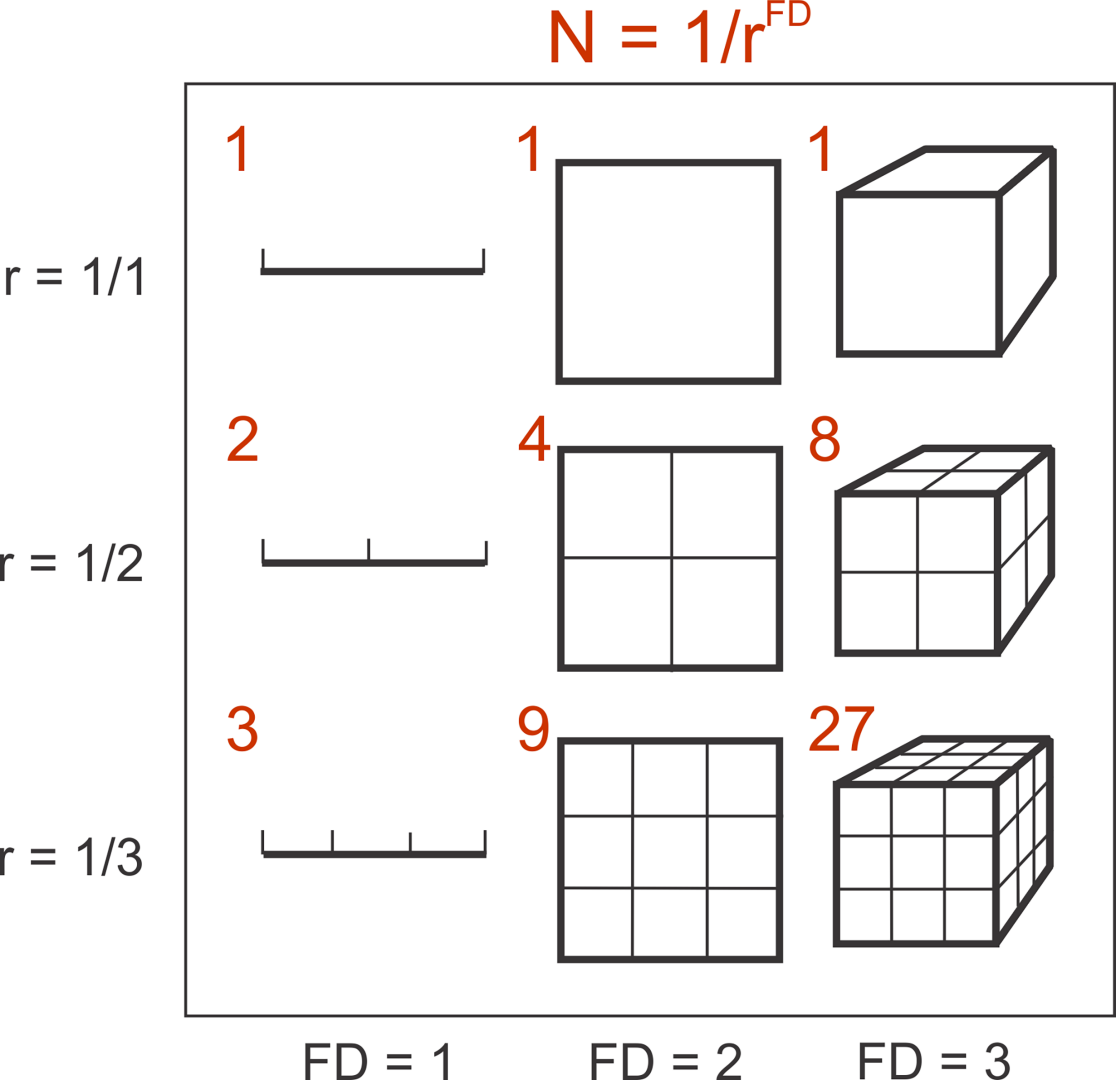
Fractal dimension. Explanation of parameters in Eq (1) causing changes in Fractal Dimension (FD).

Mandelbrot (1977) introduced fractal geometry to describe highly complex forms that are characteristic of natural phenomena such as coastlines and landscapes [21]. Mandelbrot and Ness (1968) extended the concept of statistical self-similarity to self-affine time series [24]. A time series is self-affine when its power-spectral density has a power-law dependence on frequency. The Fractal Dimension (FD) as a measure of geometric complexity can be derived mathematically for self-similar objects by Eq (1):

FD=log(N)log(1r) (1)

where N represents an object of N parts scaled by r as shown for three simple objects in Fig 1.

There are several approaches to estimate the FD of an object. The von Koch curve is an example of exactly self-similar fractals. However, natural scenes are only statistically self-similar at a finite range of scales [25–26], meaning that they are more adequately described by self-affine fractals [27]. The two-dimensional function of an image intensity surface is a fractal Brownian function [28] the fractal dimension of which can be determined either directly from the image intensity surface or from the Fourier power spectrum. These methods differ in the way they approximate the quantity N_r_ in Eq (1), but they are similar in that they use some version of the statistical relationship between the measured quantities of an object and the step sizes to derive the estimate of FD (for details see [23]).

Indeed, the dimension of a self-affine fractal is always calculated using a unique equation, which includes the value of h (Hurst coefficient) but this Hurst coefficient is determined using an equation that changes the type of self-affine fractal identified. The choice of a good equation for calculating h is often imposed by the value of the slope of the power spectrum (α) obtained through Fourier or wavelet decomposition [26–27].

Here, we will be using the Fourier technique (Power Spectrum) derived from a surface [26–28] to calculate the FD of an image. It can be shown that the Fourier power spectrum P(f), as the spectral density of a fractal Brownian function is proportional to f^(−2h−1)^, where h = 2 − FD_profile_ and f is frequency. The fractal dimension of the profile (FD_profile_) is obtained from the slope of the regression line of the log-log plot of P(f) *versus* f. The FD of a surface is computed as *FD* = FD_profile_ + 1. Detailed descriptions of the steps required to perform a spectral analysis for fractal applications can be found in Peitgen and Saupe (1988) [26] and in Turcotte (1992) [29].

We implemented an algorithm proposed by Andre Dauphine [27] in Mathematica 11.01 to calculate the fractal dimension of an image using the power spectrum. This algorithm uses Daubechies wavelets to calculate the energy spectrum that is equivalent to the Fourier decomposition power spectrum.

It is important to note that Juliani et al. [20] whose work inspired our analysis used the Box counting method for determining FD, which is the method of choice when analysing self-similar, but not self-affine, scenes [27–28,30]. In contrast, our method is the adequate one for analysing natural scenes, which are self-affine of which the self-similar fractals are subset. (e.g. [26, 28]). The results we present here, therefore, also apply to self-similar fractals.

### Acquisition and analysis of natural scene material

We recorded panoramic scenes at two different outdoor locations at the Australian National University’s campus field station simultaneously (location 1, location 2), using two “RICOH TETHA” 360 degree cameras at 0.2 frames per second (5 second intervals) and at three different times of day for durations of 13, 43 and 73 minutes. We recorded in addition 13 minutes of panoramic scenes with the same method for three different locations (Football field—location 3, City–location 4, Hill–location 5). We subsequently removed image parts below the horizon, which resulted in image sizes of 2048 × 512 pixels and converted images to grey level before determining the FD of these image time series. We calculated rotational Image Difference Functions (rotIDF) of the same image material by rotating images against themselves (auto-rotIDF) or against each other (rotIDF) using the Matlab circshift function (MathWorks, Natick, USA) or the Mathematica 11.01 program and by determining the root mean squared pixel difference for each pixel shift across the whole image (for details see [4,13]).

### Determining the orientation distribution in images

We consider a grayscale image as a two dimensional function *f*:*R*^2^ → *R* so that *f*(*x*,*y*) gives the pixel value at position (*x*,*y*).

For a function (*x*,*y*), the gradient of ∇*f* at coordinate (*x*,*y*) is defined as the two dimensional vector:

$\nabla f=\left(\frac{\partial f}{\partial x},\frac{\partial f}{\partial y}\right)=(f_{x},f_{y})$ so that the discrete derivatives of an image with function *f*(*x*,*y*) over a distance of one or two pixels are given as:

*f*_*x*_ = *f*(*x* + 1,*y*) − *f*(*x*,*y*), if Δ*x* = 1 or $f_{x}=\frac{f(x+1,y)-f(x-1,y)}{2}$, Δ*x* = 2

*f*_*y*_ = *f*(*x*,*y* + 1) − *f*(*x*,*y*), if Δ*y* = 1 or $f_{y}=\frac{f(x,y+1)-f(x,y-1)}{2}$, Δ*y* = 2, (see [31]).

The magnitude of the mean gradient vector is given by:

$$\|\nabla f\|=\sqrt{{f_{x}}^{2}+{f_{y}}^{2}}$$

and its direction or orientation by:

$$\theta ={tan}^{-1}(\frac{f_{y}}{f_{x}}).$$

Next, we consider an image as a matrix A_m×n_ so that each pixel *a*_*ij*_ has a gradient vector ${\vec{u}}_{ij}$ with direction ${\vec{\theta }}_{ij}$.

The resultant gradient vectors of all pixels in *x*−direction and in *y*−direction are:

$\vec{U}=\sum_{i,j}\left|{\vec{u}}_{ij}.\cos{\vec{\theta }}_{ij}\right|$ and $\vec{V}=\sum_{i,j}\left|{\vec{u}}_{ij}.\sin{\vec{\theta }}_{ij}\right|$, respectively. Thus, we can calculate the resultant gradient vector *T* and its orientation for any image as follows:

$$\|T\|=\sqrt{U^{2}+V^{2}}\;,\;\alpha ={tan}^{-1}\left(\frac{V}{U}\right).$$

The resultant vector of the gradient and its orientation represents the orientation statistics of images in such a way that its orientation is not affected by the number of borders or the contrast of images, but that its norm increases with increasing numbers of borders and increasing contrast (see below).

## Results

### Fractal dimension (FD), location and the dynamics of natural scenes

**Fig 2:**
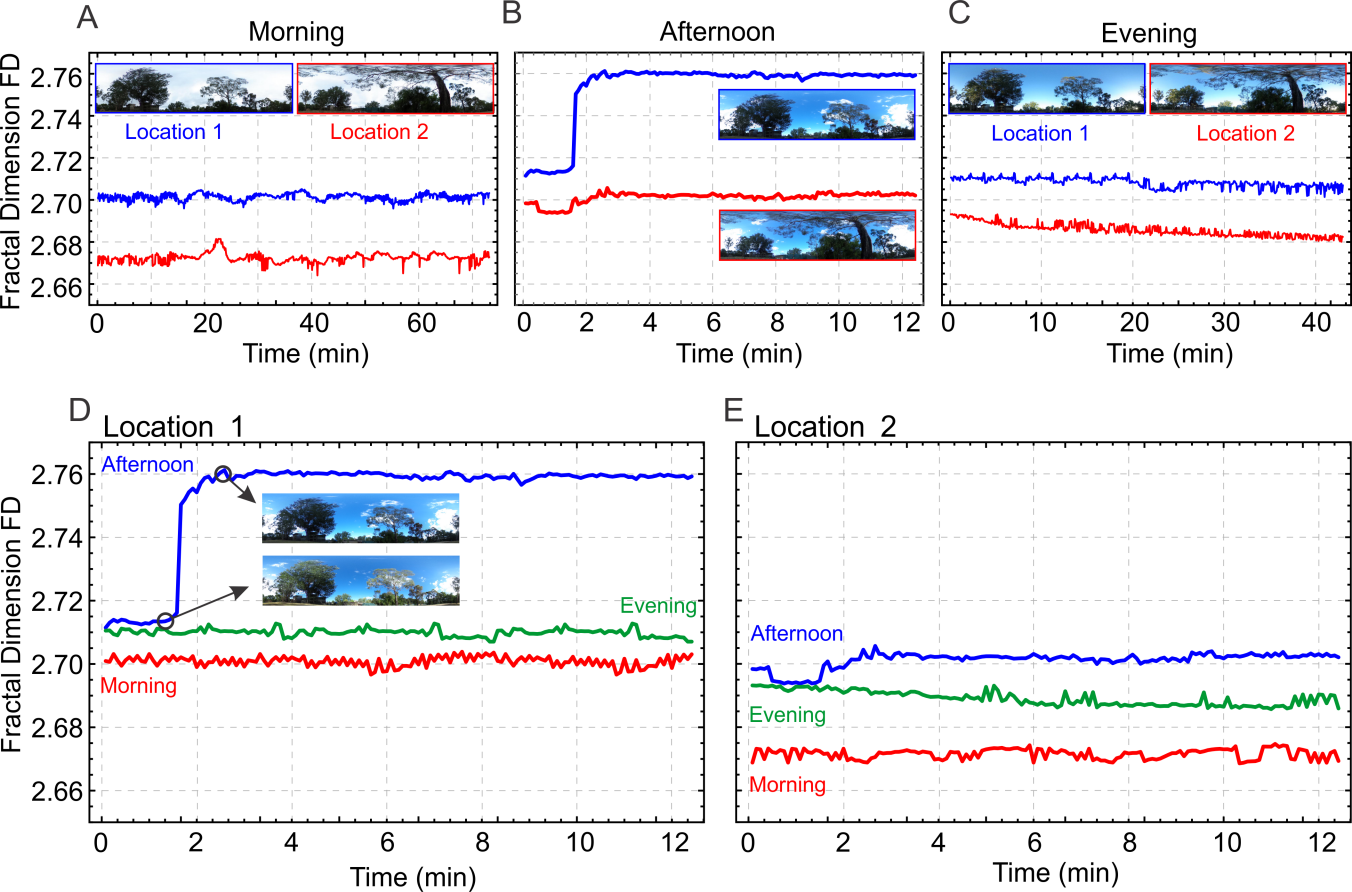
The fractal dimension (FD) of natural scenes. Panels show the FD over time of panoramic scenes at two locations (see inset images) that were 15 m distance from each other within the same landscape. The FD over time is shown for 73 min in the morning (**A**), for 13 min in the afternoon (**B**) and for 43 min in the evening (**C**). (**D**) and (**E**) show for each location the time course of FD over 13 min at three times of the day (morning, afternoon and evening). Note that large and rapid changes of FD like that shown for location 1 in B and D are caused by the movements of clouds that cover and uncover the sun with concomitant changes of reflection from foliage and of shadow contours. Image acquisition at 0.2 fps.

Before investigating the relationship between the FD and the navigational information content of natural scenes we were interested in finding out (a) how comparable these natural scenes are with the synthetic ones used by Juliani et al. (2016) [20], (b) whether different locations in the natural world show significant differences in FD and (c) how changes in illumination and the movements of clouds and shadows affect the FD of these scenes. We analysed the dynamics of the FD in image series that were recorded at two locations that were about 15 m apart in the same landscape simultaneously at three different times of day (morning, afternoon and evening). Fig 2A–2C shows the FD over time for the two locations during the morning (Fig 2A), the afternoon (Fig 2B) and the evening (Fig 2C). Short sequences of the time course of FD for each location at different times of the day are shown in Fig 2D and 2E. We find that the FD changes during the day, due to changes in illumination, but that location 1 has a consistently larger FD (2.70–2.76) compared to location 2 (2.66–2.70).

We conclude that neighbouring places in the natural world do differ in their fractal dimension, but that changes in illumination caused by the movement of the sun, shadows, wind-driven vegetation and clouds can cause large changes in FD. We will return later to the question whether the FD of a scene could be a unique identifier of location.

### Fractal dimension (FD) and the navigational information content of natural scenes

**Fig 3:**
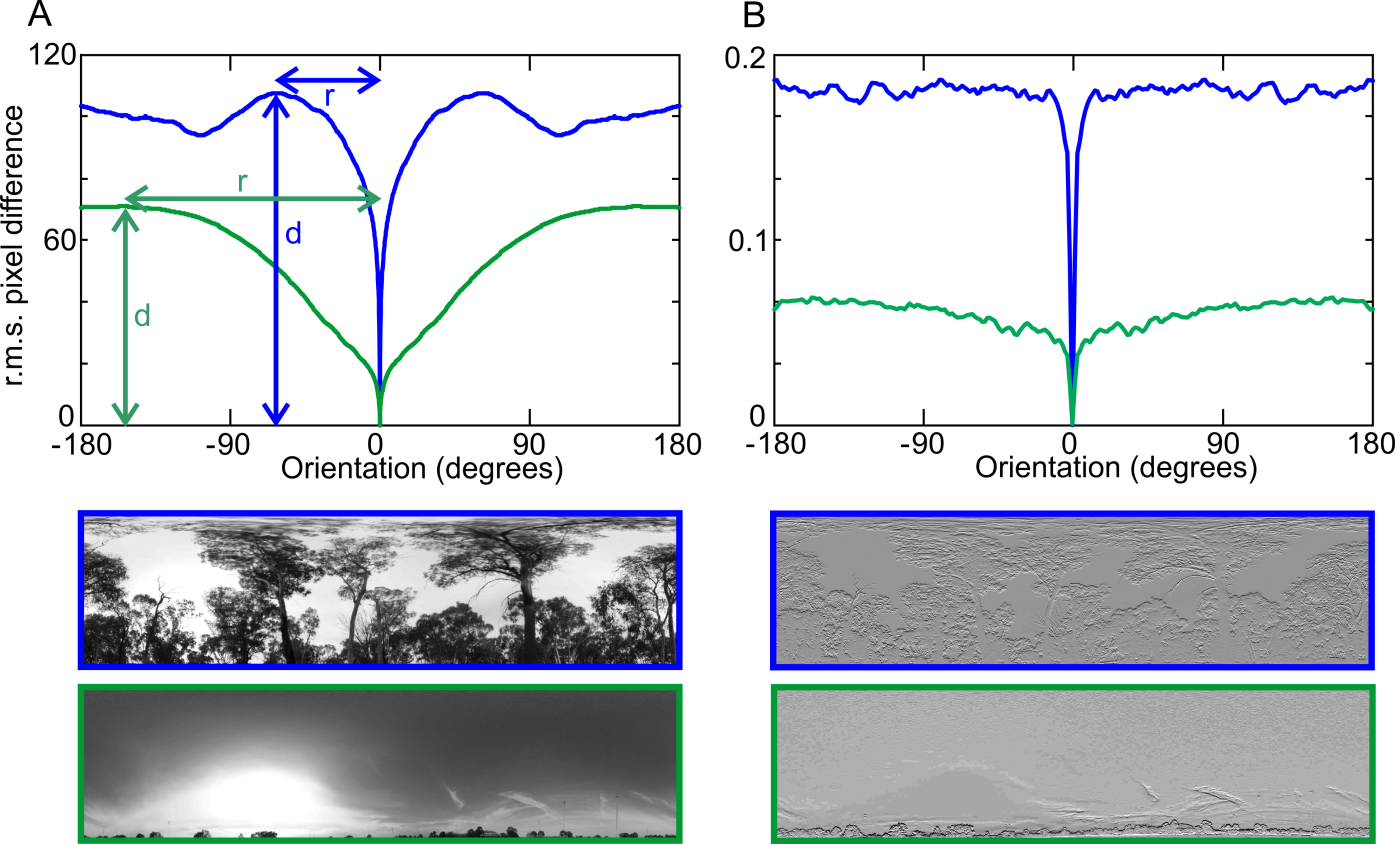
The rotational image difference function (rotIDF) as a quantitative measure of the navigational information content of natural scenes. (**A**) Two functions for the two scenes at the bottom show the r.m.s. pixel differences at different steps of rotation of each image against itself. Note differences in the depth (d) and the radius (r) of the functions and how both depend on the spatial frequency content of scenes. (**B**) The difference in rotIDF depth between different scenes is maintained after local contrast normalization. (see [4] for a quantitative analysis).

Our aim here is to investigate whether differences in the fractal dimension of views reflect differences in the navigational information they provide. In the most general sense, panoramic views provide information on orientation or compass direction because image differences exhibit a clear minimum at the reference location and increase consistently with rotation relative to the orientation of a reference view (Fig 3, rotational image difference function, rotIDF, [3–4]). This information can in principle be accessed through gradient descent in image distances (DID), one of the approaches used in holistic guidance [3,32–33]. How successfully this can be done depends on the depth (d) relative to the background noise of the function created by false minima and on the extent of its angular catchment area (r) (Fig 3A). For the purpose of this analysis we take the depth of rotIDFs as a quantitative measure of the navigational information provided by a given scene, noting firstly, that this depth (as defined in Fig 3A) depends on the feature content of scenes, being shallower in feature-less environments (compare blue with green scene, Fig 3A) and secondly, that this property is independent of image pre-processing, such as local contrast normalization (Fig 3B).

**Fig 4:**
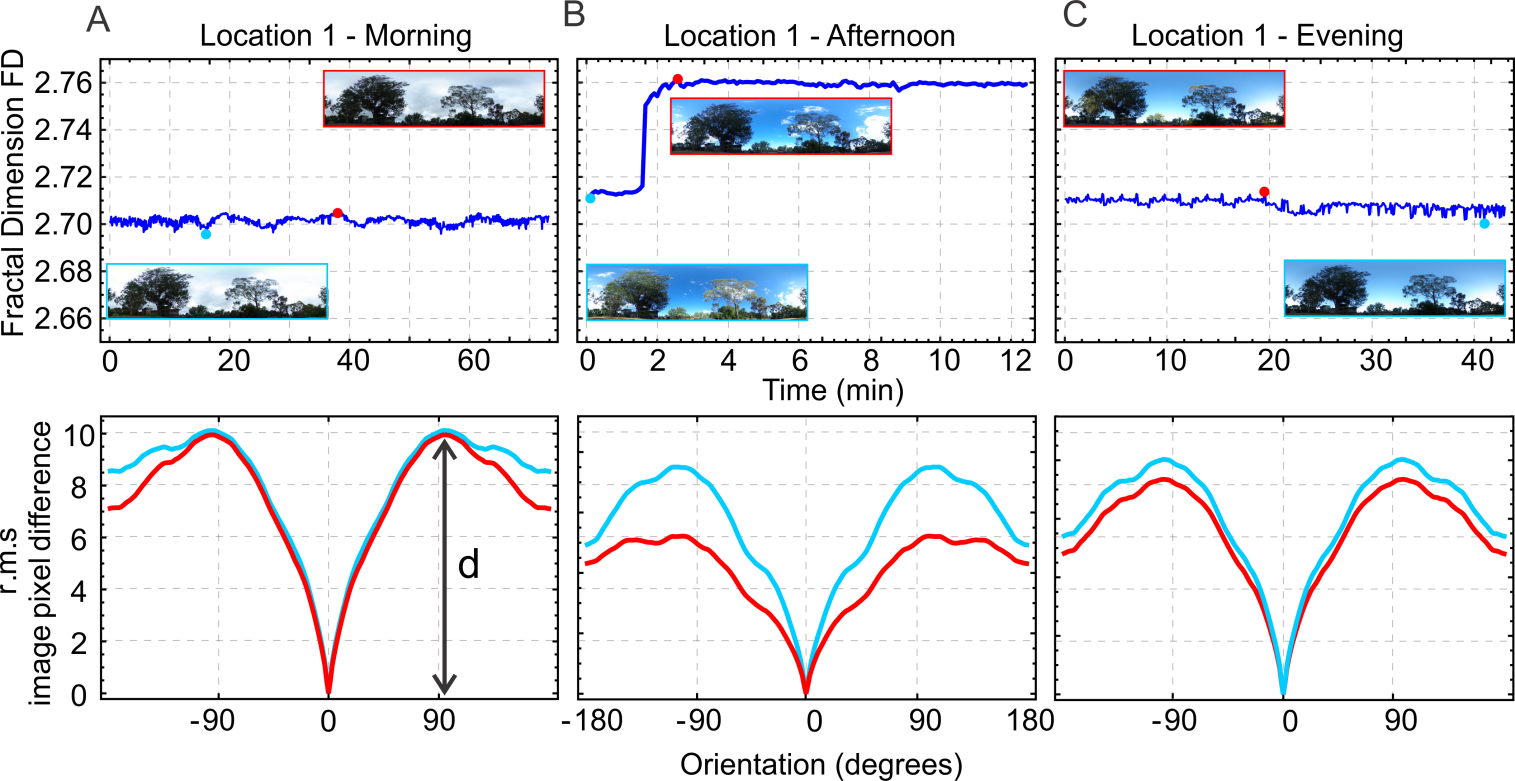
**The relationship between the Fractal dimension (FD) (top row) and the depth of the rotational image difference function (rotIDF) (bottom row)**. Shown are the rotIDFs for the same scene (location 1) when the Fractal Dimension (FD) is maximum (red) and minimum (blue) in the morning (**A**), the afternoon (**B**) and the evening (**C**). Note that there appears to be an inverse relationship between FD and the depth (d) of the rotIDF.

In a first step, we determined the depth of the rotIDF (lower panels in Fig 4) in panoramic views at location 1 for the minimum (light blue) and the maximum FD (red) in image sequences recorded at different times of day (top panels in Fig 4). The example suggests that the temporal changes in FD of a scene are reflected in changes of the depth of the rotIDF. However, given the feature dependence of the rotIDF, as demonstrated in the example shown in Fig 3, it seems counter-intuitive that at times when a scene has a high FD due to changes in illumination (marked red in Fig 4B) the depth of the rotIDF is smaller, compared to the time in which the scene has a low FD (marked light blue in Fig 4B).

**Fig 5:**
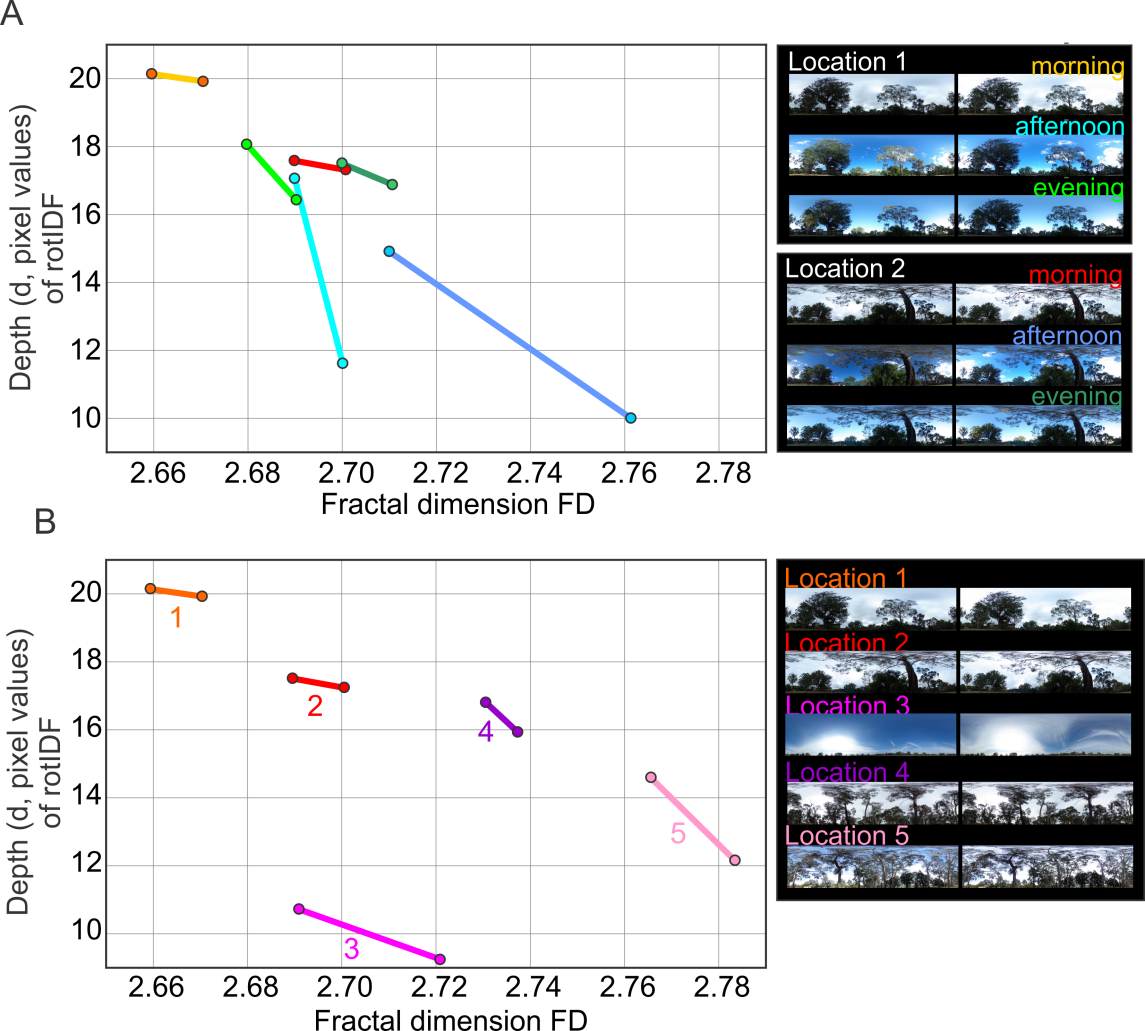
The depth of the rotational image difference function (rotIDF) is inversely related to the fractal dimension (FD) of natural scenes. (**A**) The depth of the rotIDF is shown for the maximum and minimum value of the Fractal Dimension in two scenes at different times of the day (as indicated on the right). (**B**) Same as A for 5 different locations at different days. Note that the strength of the inverse relationship or slope between the depth of the rotIDF and the Fractal Dimension is scene-specific and depends on the variance of pixel values and the contrast of images (see text for details).

This inverse relationship between FD and rotIDF depth holds for variations within (Fig 5A) and across (Fig 5B) every scene we examined. We also find that this relationship is non-linear with the same FD being associated with very different values of the rotIDF depth in different scenes (e.g. compare Location 2 and 3 in Fig 5B). We conclude that there is indeed a relationship between FD and the navigational information provided by natural scenes, but that this relationship is scene specific and affected by changes in illumination. Furthermore, different scenes can have very similar FDs (compare scene 2 and 3 in Fig 5B).

**Fig 6:**
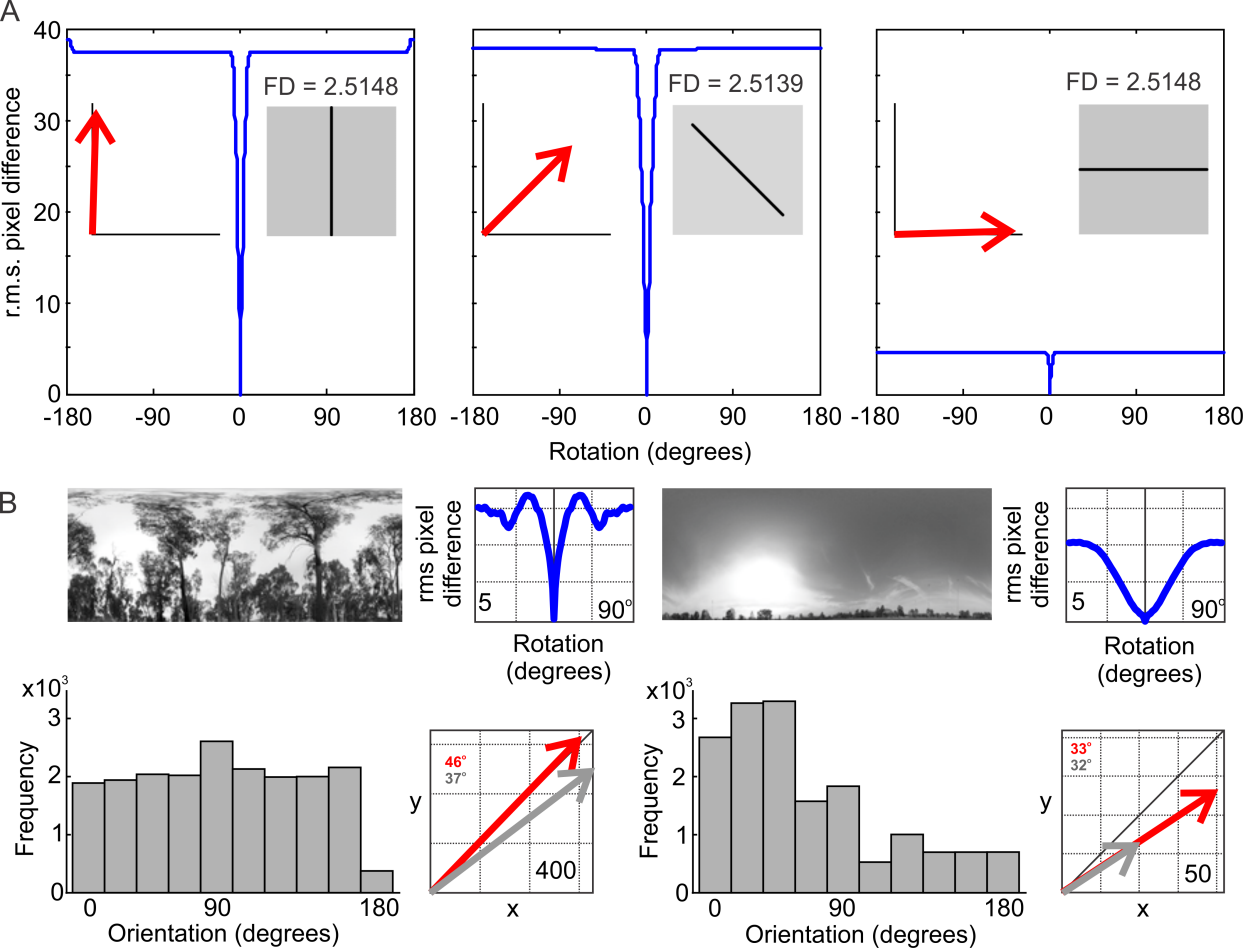
The Fractal dimension of images is independent of edge orientation while the depth of the rotational image difference function is affected by edge orientation. (A) Three images that have the same Fractal Dimension in the case of the horizontal and vertical lines and a slightly smaller FD value in the case of the 45° line (The FD is nearly independent of the orientation of the surface [28]). Blue lines show the rotIDFs for the same three images. The small remaining modulation of the rotIDF for the horizontal line is an artefact due to the rounded ends of the line. (B) The orientation distributions for location 5 (left panels) and location 3 (right panels) together with the rotIDFs of the two scenes (blue lines) and the resultant vectors of the two distributions for grey level images (red arrows) and contrast normalized images (grey arrows). Colour-coded vector orientation is given in the figures. The histograms show the orientation distributions from horizontal (0°, 180°) to vertical (90°) with bin width 18°. Note the difference in the mean direction and in the length of the resultant vectors.

We begin explaining this curious result by noting that the rotIDF depends on the orientation of edges in visual scenes, while the FD does not. We illustrate this fact for the simple case of an image of a single line in Fig 6A, while the FD of the line image is the same, regardless of whether the line is oriented vertically, at 45° or horizontally, the rotIDF depth is large for the vertical and 45° orientation, and zero for the horizontal line, as shown by the orientation of the resultant vector (red arrows in insets, Fig 6A). Note that the small remaining modulation of the rotIDF for the horizontal line is an artefact due to the rounded ends of the line. Fractal dimension is thus not affected by the distribution of orientated borders in an image, while the navigational information is (provided border contrast is the same). The inverse relationship between FD and the rotIDF may thus be caused by systematic differences and changes in the orientation statistics of natural scenes.

We demonstrate how the orientation statistics of natural scenes affects the properties of the rotIDF for two extreme examples of our data set in Fig 6B: the orientation distribution of location 5 (grey histogram, left panels, Fig 6B) is rather uniform with a peak at vertical orientations (90°), the orientation of the resultant vector is slightly larger than 45° and its length is 1600. The depth of the rotIDF for this scene is about 17 (left panel blue line in Fig 6B). The orientation distribution of location 3, in contrast (right panels, Fig 6B), is skewed towards more horizontal orientations, the resultant vector orientation is about 30° and much shorter at about 200. The rotIDF depth for that scene is smaller. Both the rotIDF depth and the length of the resultant vector depend on contrast, but the differences between the two scenes persist after contrast normalization (grey arrows in Fig 6B). The change in resultant vector orientation for location 5 (left panels Fig 6B) is probably due to reduced gradients after contrast normalization for directions diagonal to the square pixel array.

### Variance and fractal dimension

**Fig 7:**
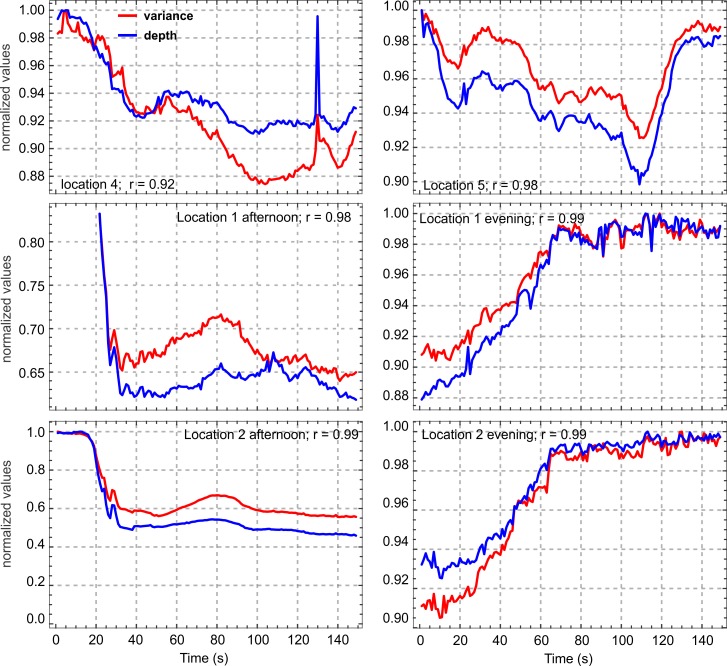
Image pixel variance and the depth of the image difference function (d) are positively correlated. This correlation is shown for normalized values of variance (red) and rotIDF depth (blue) for six different time series of different scenes and at different times of the day. Correlation coefficients and scene origins are given inside the panels.

In most of our experimental data we found a strong correlation (more than 90% and up to 99% in some cases) between the variance of pixel values and the depth of the rotIDF (Fig 7). Taking an image to be a random variable of pixel values (0–255 in our 8-bit images) then variance is the average of the squared differences of pixel values from their mean. Pixel variance may thus be a property of images that may explain the relationship between the depth of the rotIDF and the fractal dimension of an image.

In this section we show that there is an inverse relationship between fractal dimension and variance. Using spectral analysis, Malamud and Turcotte (1999) [34] provided proof that for a self-affine discrete time series y_n_, n = 1,2,3,…,N, the variance is given by

$$V(N)=\left\{ \begin{array}{l} (\frac{A}{1-\beta })[{\left(\frac{1}{2}\right)}^{1-\beta }-{\left(\frac{1}{N}\right)}^{1-\beta }]\;\mathrm{if}\;\beta <1\\ (\frac{A}{1-\beta })[{\left(2\right)}^{\beta -1}-{\left(N\right)}^{\beta -1}]\;\mathrm{if}\;\beta >1 \end{array}\right.$$

which can also be expressed in the following way:

$$V(N)=\left\{ \begin{array}{l} (\frac{A}{1-\beta })[{\left(2\right)}^{\beta -1}-{\left(N\right)}^{\beta -1}]\;\mathrm{if}\;\beta \neq 1\\ \;A(\ln(\frac{N}{2}))\;\mathrm{if}\;\beta =1 \end{array}\right.$$ (2)

with A a positive constant and β the persistence of the time series, a measure of how well adjacent values of a time series are correlated. This correlation is absent in white noise.

From Voss (1985) [35] the relationship between β, the Hausdorff exponent Ha and fractal dimension FD is given by

β=2Ha+1=5−2FD (3)

Now, if we take an image I_m×n_ (in the matrix form of an image) as a self-affine object with fractal dimension FD, then with substitution (3) in (2), we can write

$$V(I_{m\times n},\;FD)=\left\{ \begin{array}{l} (\frac{A}{-4+2FD})[{\left(2\right)}^{4-2FD}-{\left(N\right)}^{4-2FD}]\;\mathrm{if}\;\mathrm{FD}\neq 2\\ \;A(\ln(\frac{N}{2}))\;\mathrm{if}\;\mathrm{FD}=2 \end{array}\right.$$ (4)

so that N = m × n is constant.

**Fig 8:**
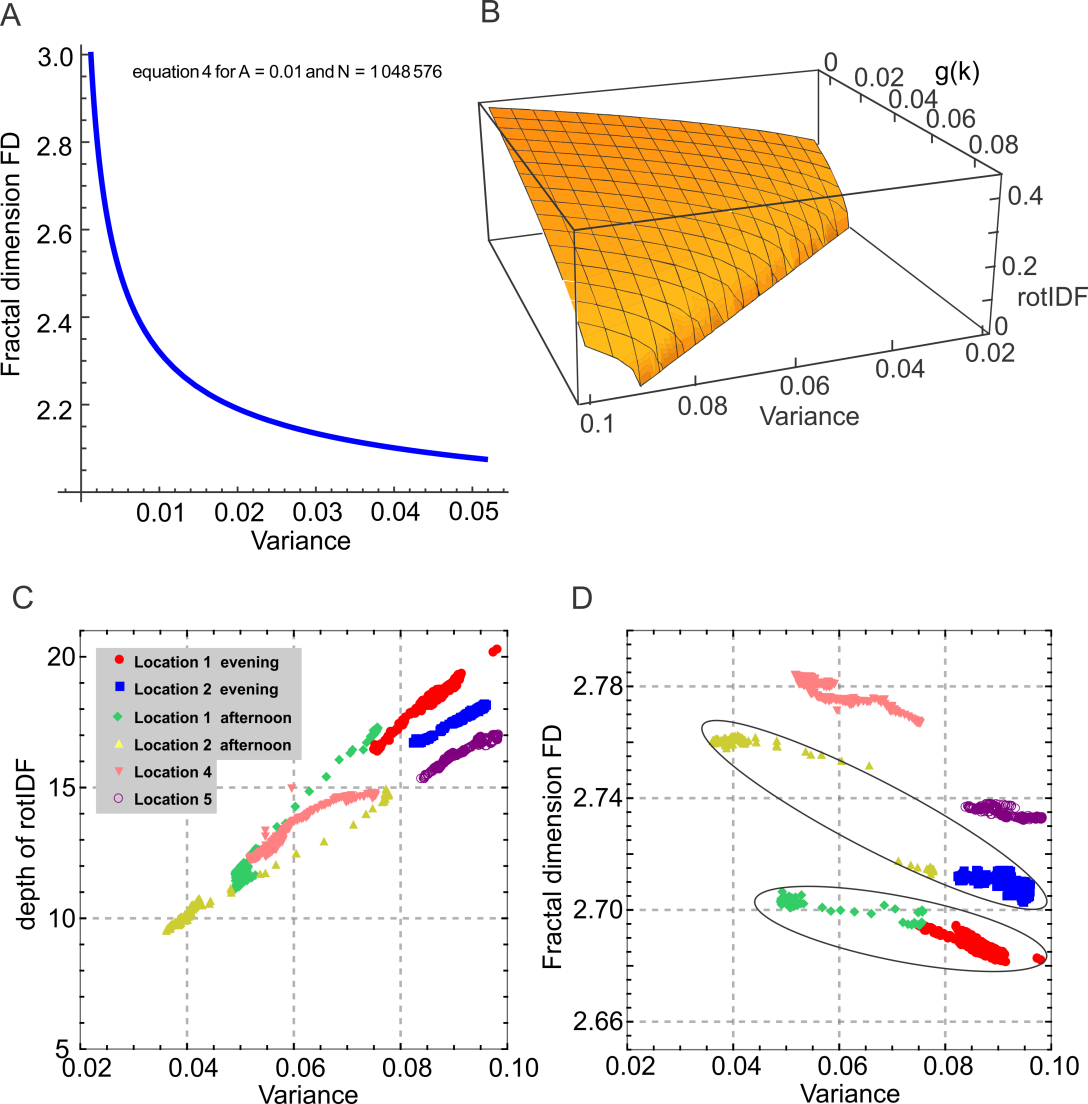
The relationship between variance, fractal dimension and the depth of the rotational image difference function. (**A**) The relationship between Fractal Dimension (FD) and Variance according to Eq (4). (**B**) Three dimensional graph of Eq (10) showing that the depth of rotIDF is an increasing function of variance and a decreasing function of g(k). (**C**) The relationship between depth of rotIDF and variance for 6 different time series of images. (**D**) The relationship between fractal dimension and variance for the same data set as shown in (B). Black lines encircle values from the same locations.

It is easy to show that in Eq (4), $\frac{dV}{dFD}<0$, so that $\frac{d}{dFD}$ is the derivative operator and V is a decreasing function of FD. According to Eq (4), Fig 8A shows that V is a decreasing function in a defined domain of an image. We conclude that variance and fractal dimension of an image are inversely related. Experimental data of 6 time series of images in 5 different locations confirm the inverse relationship between FD and variance (see Fig 8D).

### Pixel variance and the depth of the rotIDF

We aim next to find the relationship between variance and the depth of the rotational image difference function. For this we define the rotIDF for a pixel matrix I_m×n_ (see [3,4,8]) as:

$$rIDF(I_{m\times n})=\sqrt{\frac{{\sum \left(I-I^{k}\right)}^{2}}{m.n}},$$ (5)

with I^k^ = I horizontally shifted by step k, 1 ≤ k ≤ n.

We can re-write Eq (5) in a different way as follows:

Consider the matrix form of an image $I_{m\times n}=\left[\begin{array}{ccc} a_{11} & \cdots & a_{1n}\\ \vdots & \ddots & \vdots \\ a_{m1} & \cdots & a_{mn} \end{array}\right]$

then

$$rIDF(I_{m\times n},k)=\sqrt{\frac{f(k)}{m.n}},$$

with

$$f(k)=\sum_{i=1}^{k}\sum_{j=1}^{m}{\left(a_{ji}-a_{j(n+i-k)}\right)}^{2}+\sum_{i=k+1}^{n}\sum_{j=1}^{m}{\left(a_{ji}-a_{ji-k}\right)}^{2}$$ (6)

Eq (6) can be used to calculate the difference between two images.

Now, suppose that $\bar{x}$ is the mean value of the matrix I_m×n_, by subtracting and adding $\bar{x}$ to Eq (6) and some simplifications we have

$$\begin{array}{l} f\left(k\right)=\sum_{i=1}^{k}\sum_{j=1}^{m}{\left(a_{ji}-\bar{x}+\bar{x}-a_{j\left(n+i-k\right)}\right)}^{2}+\sum_{i=k+1}^{n}\sum_{j=1}^{m}{\left(a_{ji}-\bar{x}+\bar{x}-a_{ji-k}\right)}^{2}=\ldots =\\ \sum_{i=1}^{k}\sum_{j=1}^{m}{\left(a_{ji}-\bar{x}\right)}^{2}+\sum_{i=k+1}^{n}\sum_{j=1}^{m}{\left(a_{ji}-\bar{x}\right)}^{2}+\\ \sum_{i=1}^{k}\sum_{j=1}^{m}{\left(a_{j\left(n+i-k\right)}-\bar{x}\right)}^{2}+\sum_{i=k+1}^{n}\sum_{j=1}^{m}{\left(a_{ji-k}-\bar{x}\right)}^{2}+\\ \left[-2\sum_{i=1}^{k}\sum_{j=1}^{m}\left(a_{ji}-\bar{x}\right)\left(a_{j\left(n+i-k\right)}-\bar{x}\right)-2\sum_{i=k+1}^{n}\sum_{j=1}^{m}\left(a_{ji}-\bar{x}\right)\left(a_{ji-k}-\bar{x}\right)\right] \end{array}$$

then

$$\begin{array}{l} \frac{f\left(k\right)}{m.n}=\frac{\sum_{i=1}^{k}\sum_{j=1}^{m}{\left(a_{ji}-\bar{x}\right)}^{2}+\sum_{i=k+1}^{n}\sum_{j=1}^{m}{\left(a_{ji}-\bar{x}\right)}^{2}}{m.n}+\frac{\sum_{i=1}^{k}\sum_{j=1}^{m}{\left(a_{j\left(n+i-k\right)}-\bar{x}\right)}^{2}+\sum_{i=k+1}^{n}\sum_{j=1}^{m}{\left(a_{ji-k}-\bar{x}\right)}^{2}}{m.n}+\\ \frac{\left[-2\sum_{i=1}^{k}\sum_{j=1}^{m}\left(a_{ji}-\bar{x}\right)\left(a_{j\left(n+i-k\right)}-\bar{x}\right)-2\sum_{i=k+1}^{n}\sum_{j=1}^{m}\left(a_{ji}-\bar{x}\right)\left(a_{ji-k}-\bar{x}\right)\right]}{m.n}=2Variance\left(I_{m\times n}\right)-2g\left(k\right);\\ g\left(k\right)=\frac{\sum_{i=1}^{k}\sum_{j=1}^{m}\left(a_{ji}-\bar{x}\right)\left(a_{j\left(n+i-k\right)}-\bar{x}\right)+\sum_{i=k+1}^{n}\sum_{j=1}^{m}\left(a_{ji}-\bar{x}\right)\left(a_{ji-k}-\bar{x}\right)}{m.n} \end{array}$$ (7)

Now we can write

$$g(k)=\frac{\sum \left(I-\bar{x})*(I^{k}-\bar{x}\right)}{m.n}$$ (8)

with the symbol “*” denoting the Hadamard product of two matrices. Using one of the properties of the Hadamard product (∑A * B = tr(A. B^t^)) we can write for g(k)

$$g(k)=\frac{tr[(I-\bar{x}).{\left(I^{k}-\bar{x}\right)}^{t}]}{m.n},$$ (9)

with "tr" being the trace of a matrix. Originally g(k) is the sum of the Hadamard product first order distance (mean) of an image and its k − rotated self or the sum of the diagonal values of multiplication of the first order distance matrix for an image and its k − rotated self. Plotted on its own, g(k) is the inverse of the rotIDF, with the rotIDF minimum coinciding with the maximum of g(k).

Thus, the new form of Eq (5) is

$$rotIDF(I_{m\times n},k)=\sqrt{2Var(I_{m\times n})-2g(k)}\;1\leq k\leq n$$ (10)

with $g(k)=\frac{tr[(I-\bar{x}).\;{\left(I^{k}-\bar{x}\right)}^{t}]}{m.n}$ or $g(k)=\frac{\sum \left(I-\bar{x})*(I^{k}-\bar{x}\right)}{m.n}=\frac{\sum (I-\bar{x})*{\left(I-\bar{x}\right)}^{k}}{m.n}=\frac{\sum H*H^{k}}{m.n};H=I-\bar{x}$

We note that Eq (10) can be proven using the basic result in statistics: for any two random variables x and y, E[(x-y)^2^ ] = E[x^2^]-E[y^2^]-2E[x.y]. Now, we consider one time series of images and suppose that in the function $z=\sqrt{x-y}$, z, x and y are rotIDF, variance and g(k). It is clear that $\frac{\partial z}{\partial x}>0,\;\frac{\partial z}{\partial y}<0$. These inequalities show that the rotIDF is directly related to variance (increasing with variance) and inversely related to g(k) (decreasing with g(k)). Therefore, the maximum of the rotIDF or its depth coincides with the minimum of g(k). Conversely, the maximum value of g(k) corresponds to the variance of the image pixel values, so that we can approximate the maximum of Eq 10 as:

$$\sqrt{2(Max(g(k))-Min(g(k)))}\approx Max(rotIDFs)\approx depth.$$

Eq (10) is well-defined for any image with dimension m×n as long as the condition g(k) ≤ Var(I(m×n)) holds. A three dimensional plot of Eq (10) is shown in Fig 8B. Note that Eq (10) can also be interpreted from the perspective of image contrast. As RMS contrast is defined as the standard deviation of the pixel intensities (the square root of variance) [36], it is proportional to variance. According to Eq (10), therefore, the depth or maximum of the rotIDF increases with variance and image contrast, while fractal dimension is inversely proportional to variance and contrast (see Fig 8C and 8D).

This theoretical proof is corroborated by the experimental data shown in Fig 5 and Fig 8C and 8D for six different image sequences at different locations. We thus conclude that the inverse relationship between fractal dimension and the depth of the rotIDF (as our measure of navigational information) is based on differences in image pixel variance that is inversely proportional to FD and proportional to rotIDF depth (Fig 8C and 8D). The clustering of FD values for different scenes in Fig 8D suggests that there may be the possibility of locations in the natural world being identifiable by their FD value distribution. However, FD variations due to changes in illumination as highlighted for site 1 and site 2 by black lines in Fig 8D can be larger than the differences between sites.

**Fig 9:**
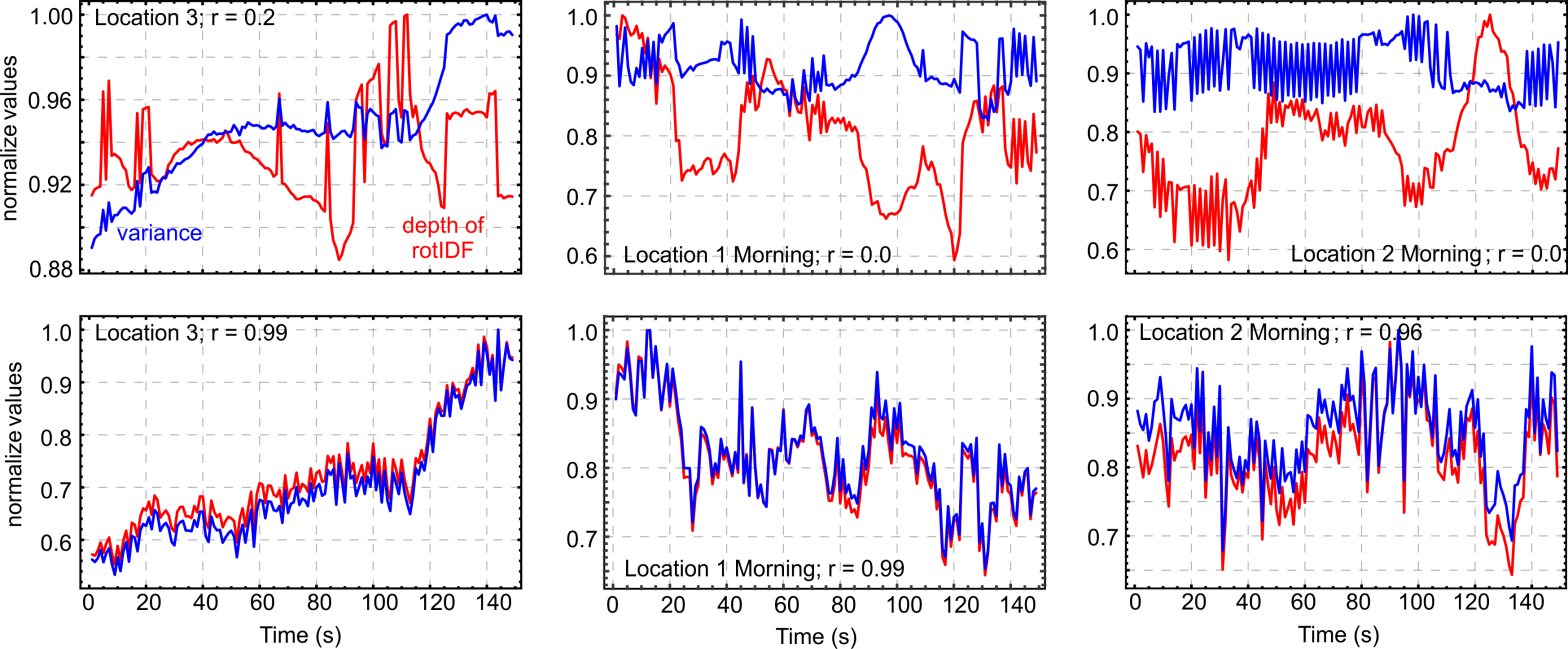
Three examples of image time series in which the correlation between depth and variance was weak or absent. The scenes were recorded at times when illumination changed dramatically due to heavy, wind-driven cloud cover which engaged the camera’s automatic gain (and shutter) control. Top panels: Time course of rotIDF depth (red) and variance (blue) of original time series. Bottom panels: Same after vertical derivate filtering which is equivalent to local contrast normalization.

Finally, we do need to add a word of caution here that the way in which images are acquired is important for this kind of analysis. In our experimental data we encountered three time series of images in which there were no or only weak correlations between rotIDF depth and variance (see top row Fig 9). Yet, the correlation is recovered by vertical derivative filtering of these data (the derivative along vertical rows of pixel values), which is equivalent to local contrast normalization (bottom row Fig 9). The scenes in question were all recorded at times when illumination changed dramatically due to heavy, wind-driven cloud cover and we suggest that this engaged the camera’s automatic gain (and aperture) control, which in turn may have affected the correlation between depth and variance.

### A special case in the relationship between rotIDF depth and fractal dimension

We are now in the position to investigate why some scenes have the same fractal dimension but different depths of the rotIDF (see Fig 5). Consider two images of the same size, for instance one with a horizontal line and one with a vertical line (Fig 6). According to Eq (4), the fractal dimension of these two images is the same and their variance should also be the same. On the other hand, if we analyse these two images (with the same fractal dimension or the same variance) using Eq (10) to calculate the depth of the rotIDF(I_m×n_,k), we see that the image with the horizontal line has a value near zero while the depth of the rotIDF for the image with the vertical line is much larger than zero.

We note firstly that the maximum value of the function rotIDF(I_m×n_,k) (Eq 10) for a constant value of variance is completely determined by the function g(k) the range of which is defined by the operator k − rotate (horizontally). Since the value of g(k) for a matrix I_m×n_ and its transpose (I_m×n_)^t^ are not equal (except for symmetrical matrixes), we conclude that the values of the rotIDF(I_m×n_,k) of these two images are not equal (see appendix). The second point to note is that in images with the same fractal dimension and different depths of the rotIDF (see for instance Fig 5B), FD as a function of rotIDF depth is constant. Since any constant function can be (non-strictly) a decreasing or an increasing function there is no conflict with the inverse relationship of fractal dimension and rotIDF depth.

## Discussion

We have shown here that the dimension of a self-affine fractal time series (a time series of natural images) is inversely related to the depth of the rotational image difference function. We have further shown that the geometric complexity of natural scenes (as measured by their fractal dimension) and the navigational information they provide (as measured by the rotational image difference function) are related through image pixel variance and therefore image contrast. The new Eq (10) thus relates navigational information to quantifiable image properties. Although the inverse relationship between fractal dimension and the navigational information provided by natural scenes holds for all the scenes we analysed, the strength of this relationship clearly differs between different scenes, to the degree that scenes with similar FD can have very different depths of the rotIDF (Fig 5A and 5B). We suggest that this is likely to be due to differences in distribution of edge orientations with similar contrast in different scenes, because fractal dimension is independent of edge orientation, while rotational image difference functions are affected exclusively by the vertical components of edges.

Our results thus appear to corroborate in a quantitative way the conclusions reached by Juliani et al. (2016) who found an inverse relationship between the fractal dimension of virtual scenes and the performance of human observers in localizing goals in virtual environments, the postulated reason being that low fractal dimension scenes offer more cues for recognizing individual landmarks, which is made intuitively clear by the views of Juliani et al.’s environments in their Fig 3 [20]. When locating a goal location humans are indeed likely to make use of individual object recognition rather than of information provided by the unsegmented panorama [18–19].

In contrast, our approach and our measure of navigational information content is inspired by concepts and experiments from insect and robot navigation research where attempts are being made to explain and implement navigational performance without the need for image segmentation or object recognition [5,37–38]. It is thus particularly interesting that our analysis and that of Juliani et al. (2016) arrive at the same, in hind-sight quite obvious, conclusion: it is much harder to identify a location in a complex, densely occupied and in the case of Juliani et al.’s virtual scenes, self-similar environment (see Fig 1B, bottom right), such as the Australian Bush, than in an open (self-affine?) landscape (Fig 1B, top left). This does not depend on the particular homing mechanism. A homing agent operating with landmark recognition would have a larger ‘recognition range’ in open habitats because distinct landmarks are visible at larger distances and an agent operating by gradient descent in image differences relative to a panoramic reference snapshot [3,33] would experience a larger range over which the gradient is detectable (see [8]).

We finally note that our analysis needs to be extended to investigating how translational image differences are affected by scene properties [4]. In contrast to the rotational image differences we investigated here, which are affected by rotational optic flow that is of the same magnitude throughout the visual field, image differences due to translation are generated by translational optic flow, the magnitude of which depends on the viewing direction relative to the direction of movement and on the distance distribution of objects in the environment [4,37–38]. We have shown here that the strength of the relationship between fractal dimension and navigational information as measured by the rotational image difference function depends on the degree to which a scene contains vertical edges that generate rotational image differences (because optic flow directions are uniform around the axis of rotation). In contrast, translational optic flow is generated by both vertical and horizontal edges so that the strength of the relationship between fractal dimension and navigational information as measured by the translational image difference function should be orientation invariant.

### Appendix

Suppose an image with one horizontal black line (I_3×3_). In matrix form $I_{3\times 3}=\left[\begin{array}{ccc} 1 & 1 & 1\\ 0 & 0 & 0\\ 1 & 1 & 1 \end{array}\right]$, and its transpose (a vertical black line) ${I^{t}}_{3\times 3}=\left[\begin{array}{ccc} 1 & 0 & 1\\ 1 & 0 & 1\\ 1 & 0 & 1 \end{array}\right]$. If we rotate I_3×3_ horizontally (for example to the right) all three rotation steps are same and the rotIDF(I_3×3_,3) = 0.The depth of the rotIDF is therefore zero. Now consider the three rotation steps for I^t^_3×3_ as $rotStep\;1=\left[\begin{array}{ccc} 1 & 1 & 0\\ 1 & 1 & 0\\ 1 & 1 & 0 \end{array}\right],\;rotStep\;2=\left[\begin{array}{ccc} 0 & 1 & 1\\ 0 & 1 & 1\\ 0 & 1 & 1 \end{array}\right]$ and $rotStep\;3=\left[\begin{array}{ccc} 1 & 0 & 1\\ 1 & 0 & 1\\ 1 & 0 & 1 \end{array}\right]$ then it is obvious that rotIDF(I_3×3_,3) > 0. This simple example shows that rotIDF(I_m×n_,k) ≠ rIDF(I_m×n_^t^,k) meaning that in the case in which the rotation operator is a horizontal shift operator, any image with more vertical contours should generate a rotational image difference function with a larger amplitude or depth compared to an image with more horizontal contours.
